# The British Medical Association in Scotland

**Published:** 1914-07-11

**Authors:** 


### The British Medical Association in Scotland.

The Council of the British Medical Association has decided to appoint a whole-time medical secretary for Scotland, with the object of making better provision for the organisation of the profession and the Association. The appointment will, it is stated, be made at the forthcoming Aberdeen meeting. The duties will begin in January 1915. They will include the secretaryship of the Scottish Committee and the carrying out of its instructions, subject to the authority of the representative body and the Council. The medical secretary will also collect, edit, and transmit to the editor of the *British Medical Journal* all reports of Scottish divisions, branches, and local medical committees, and, so far as necessary, of other Scottish medico-political bodies. The salary attaching to the new post will be £600. Another new departure in the organisation of the British Medical Association in Scotland is the creation of a deputy-chairmanship of the Scottish Committee, which will require the sanction of a special representative meeting. This step is rendered necessary by the growing work of the Scottish Committee, which makes it often very difficult for the chairman to attend both the committee meetings in Scotland and Council or committee meetings in London as well.

